# A prognostic tool for pulmonary collapse: nomogram-based prediction of 28-day mortality

**DOI:** 10.3389/fdata.2026.1728498

**Published:** 2026-07-09

**Authors:** Xinming He, Wenchong Yu, Yuling Li, Ao Ma, Zhichao Meng, Jiehao Zhu, Minghui Tan, Xiaodong Zhao, Mu Chen

**Affiliations:** 1 Department of Pathology, The First Affiliated Hospital of Guangzhou Medical University, Guangzhou, China; 2 Department of Orthopedics, Foshan Fosun Chancheng Hospital, Foshan, China; 3 Foshan Clinical Medical School of Guangzhou University of Chinese Medicine, Guangzhou, China; 4 Department of Orthopedics, The First Affiliated Hospital of Jinan University, Guangzhou, China; 5 Department of Respiratory, Guangzhou Eighth People's Hospital of Guangzhou Medical University, Guangzhou, China; 6 Department of Respiratory Medicine, Guangzhou Hospital of Integrated Tradtional Chinese and Westem Medicine, Guangzhou, China

**Keywords:** MIMIC database, mortality, nomogram, prognosis, pulmonary collapse

## Abstract

**Background:**

Pulmonary collapse is a common and serious respiratory condition, but there is no dedicated bedside tool to estimate prognosis. This study aimed to develop a nomogram to predict 28-day mortality in patients with pulmonary collapse.

**Methods:**

We extracted data for patients with pulmonary collapse from MIMIC-III, identified predictors using regression analyses, and used MIMIC-IV for temporal validation. We then built a nomogram based on the selected predictors. Model performance was evaluated using the area under the receiver operating characteristic curve (AUC), AUC comparisons using the DeLong test, reclassification (NRI and IDI), calibration (calibration curves, calibration slope, and Brier score), and decision curve analysis (DCA).

**Results:**

A total of 4,088 patients with pulmonary collapse were included in the study. Logistic regression analysis identified twelve independent predictive factors associated with 28-day mortality: age (OR = 1.01, *P* =0.040), married status (OR =0 .63, *P* =0 .040), Glasgow Coma Scale score (OR = 0.94, *P* = 0.02), creatinine (OR = 0.82, *P* = 0.03), chloride ions (OR = 0.85, *P* = 0.03), sodium ions (OR = 1.19, *P* = 0.02), blood urea nitrogen (OR = 1.02, *P* < 0.001), white blood cell count (OR = 1.04, *P* < 0.001), heart rate (OR = 1.02, *P* = 0.02), respiratory rate (OR = 1.05, *P* = 0.03), temperature (OR = 0.54, *P* < 0.001), and metastatic cancer (OR = 6.66, *P* < 0.001). The nomogram showed moderate discrimination and consistently higher AUC than Age+Gender, SOFA, and SAPSII across cohorts.

**Conclusion:**

This study identified factors associated with 28-day mortality in patients with pulmonary collapse and developed a nomogram for early risk stratification.

## Introduction

Pulmonary collapse is a critical and potentially fatal respiratory condition caused by various factors that lead to lung tissue compression, resulting in reduced lung capacity and impaired gas exchange (Hedenstierna and Rothen, 2012). This can occur due to obstructive damage from mucus, bronchial lesions, or foreign bodies that disrupt the continuity of alveolar airways, as well as the accumulation of fluid, pneumothorax, chest wall masses exerting direct pressure on the lungs, or a reduction in surfactant production (Woodring and Reed, 1996). Occurrences of pulmonary collapse in hospitals are relatively common, particularly among critically ill patients (Leong et al., 2017; Du et al., 2017). Those with respiratory diseases, such as lung infections and chronic obstructive pulmonary disease (COPD) are at a higher risk of developing pulmonary collapse (Leong et al., 2017; Du et al., 2017). Pulmonary collapse can rapidly lead to symptoms such as difficulty breathing, chest pain, low blood pressure, and impaired lung function, potentially posing a life-threatening risk (Imran and Eastman, 2017).

Despite advancements in the diagnosis, surgical methods, and treatment of complications associated with pulmonary collapse, the mortality rate among these patients remains very high (Peroni and Boner, 2000). These patients often have underlying pulmonary diseases and poor overall health, which contribute to higher onset and mortality rates, thereby intensifying treatment challenges and the risk of mortality with each episode (Galvez et al., 2015). Some patients experience delays in treatment due to a lack of recognition of early symptoms, resulting in disease progression to irreversible stages (Gillett et al., 2021). Moreover, infections, bleeding, muscle damage, and post-operative pain following surgical interventions are critical factors contributing to increased mortality rates (Ozyilmaz and Kaya, 2012). A thorough understanding of mortality-related factors in patients with pulmonary collapse is essential for optimizing ICU treatment and care, ultimately minimizing the death toll as much as possible.

Predicting the mortality rate of patients with pulmonary collapse remains a complex challenge. Although Sequential Organ Failure Assessment (SOFA) and Acute Physiology and Chronic Health Evaluation II (APACHE II) scores are somewhat helpful in assessing the condition of ICU patients, they lack specificity and sensitivity in evaluating the clinical practices and prognosis of pulmonary collapse patients, thus failing to effectively guide the treatment of such patients in clinical settings (Beigmohammadi et al., 2022). Nomograms, as visual tools, offer potential in clinical management and prognosis assessment (Lamberink et al., 2017; Fang et al., 2022). However, nomograms that predict the risk factors associated with mortality in pulmonary collapse patients have received little attention. To integrate different risk factors into a comprehensive predictive model, our study aims to construct a nomogram specifically designed to predict the 28-day mortality rate of pulmonary collapse patients, thereby guiding clinical practice.

## Materials and methods

### Data source

The data used in this study were obtained from MIMIC-III and MIMIC-IV. MIMIC-III includes adult ICU admissions at Beth Israel Deaconess Medical Center between 2001 and 2012 (Johnson et al., 2016), and MIMIC-IV includes admissions from 2008 to 2019 (Johnson et al., 2023). Both databases are de-identified public datasets, so informed consent was waived. All study personnel completed required training and had authorized access to the MIMIC databases (Certification number: 40269495).

### Patients and variables

**Figure 1:**
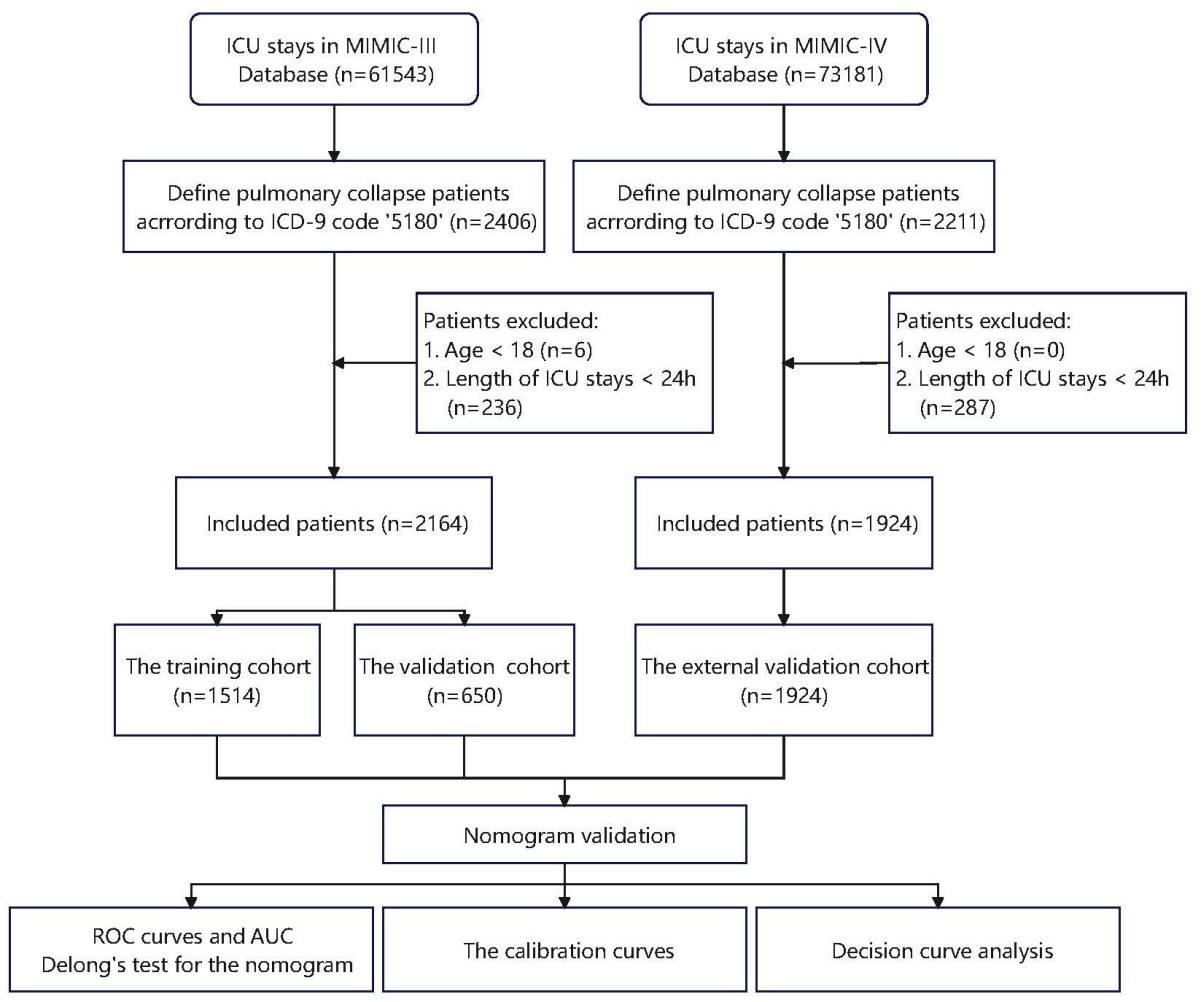
Study flowchart.

Data were extracted using SQL in DBeaver Community (version 22.2; DBeaver Corp, New York, NY, USA). We identified ICU patients with pulmonary collapse using a single ICD-9-CM code, 518.0 (stored as 5180 in MIMIC-III), and did not combine other diagnosis codes for cohort entry. As a state code, ICD-9-CM 518.0 may include heterogeneous entities (atelectasis, airway obstruction, compression, or other causes) with related but non-identical mechanisms (Quan et al., 2005; Mullett et al., 2012). We excluded patients who were (1) younger than 18 years or (2) admitted to the ICU for less than 24 h. The patient flowchart is shown in Figure 1. Candidate predictors were defined using data from the first 24 h after ICU admission. Variables included: (1) demographics: age, gender, race, marital status; (2) severity scores: Glasgow Coma Scale (GCS), Sequential Organ Failure Assessment (SOFA), Simplified Acute Physiology Score II (SAPSII), and Acute Physiology Score III (APSIII); (3) vital signs: urine output, heart rate, blood pressure, respiratory rate, temperature, peripheral oxygen saturation; (4) laboratory findings: anion gap, bicarbonate, creatinine, chloride, glucose, hematocrit, platelet count, potassium, partial thromboplastin time (PTT), international normalized ratio (INR), prothrombin time (PT), blood urea nitrogen (BUN), white blood cell count (WBC); and (5) comorbidities: heart disease, chronic lung disease, diabetes, liver disease, peptic ulcer, AIDS, lymphoma, solid tumor, metastatic cancer, and rheumatoid arthritis. The primary outcome was 28-day mortality.

### Statistical analysis

Missing data were handled using multiple imputation with the “mice” package in R (version 4.0.3; R Foundation for Statistical Computing, Vienna, Austria). Variables with >20% missing values were excluded (none met this threshold). We performed 20 imputations using predictive mean matching, and the imputed datasets were combined using Rubin's rules. Continuous predictors were entered in their original clinical units and were not standardized; odds ratios therefore reflect a one-unit increase in the original scale. Because the objective was baseline risk prediction at ICU , treatment variables after admission (e.g., post-baseline ventilator adjustments or subsequent therapeutic interventions) were not used as predictors. Patients with pulmonary collapse from MIMIC-III were randomized into a training or validation group (7:3); 1,514 patients in the training group were used to build the nomogram model, whereas 650 patients in the validation group were used to validate the model. The cohort extracted from MIMIC-IV was used as an external validation cohort. While MIMIC-III and MIMIC-IV are temporally distinct, both originate from the same institution (Beth Israel Deaconess Medical Center), thus representing quasi-external validation rather than fully independent validation across different healthcare systems. Categorical variables were expressed as frequencies and proportions, and comparisons were made using the χ^2^ test or Fisher's exact test. Continuous variables were identified by the Shapiro–Wilk test, and normally and non-normally distributed continuous variables were expressed as mean and standard deviation, median and interquartile distance, respectively.

**Figure 2:**
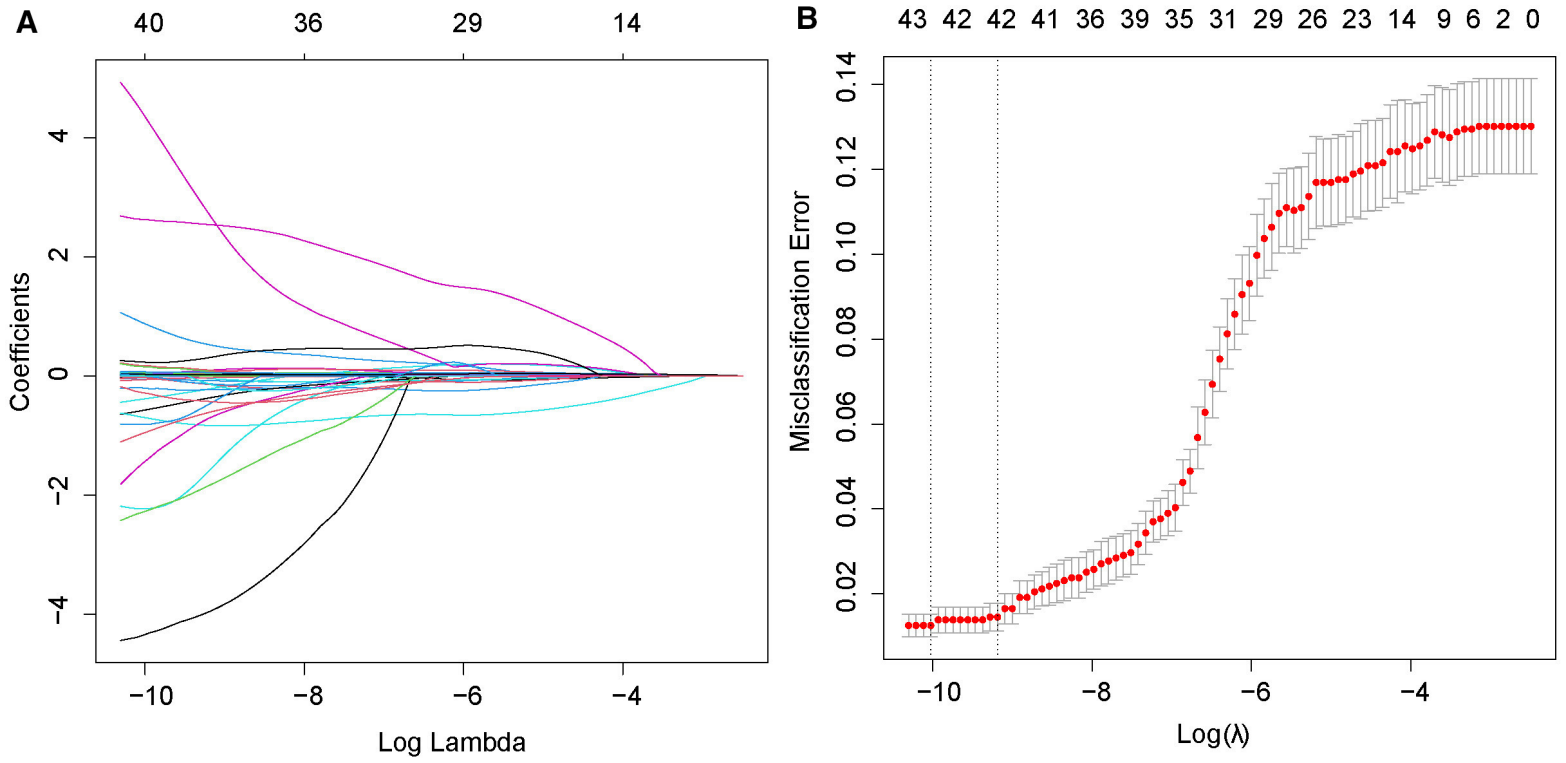
LASSO variable-selection process for 28-day mortality modeling in patients with pulmonary collapse. **(A)** Coefficient profiles of candidate predictors across penalty values. **(B)** Ten-fold cross-validation curve used to select lambda (minimum-criterion lambda = 0.032).

Variable selection was performed using LASSO (Least Absolute Shrinkage and Selection Operator) regression to handle multicollinearity and select predictors associated with 28-day mortality. The optimal regularization parameter (lambda) was determined via 10-fold cross-validation using the lambda.1se (lambda = 0.032). Coefficient paths are shown in Figure 2A, and the cross-validation curve in Figure 2B. Selected variables were then entered into multivariable logistic regression for final coefficient estimation. The selected variables were subjected to multivariable logistic regression analysis, and the statistically significant variables were finally identified, and the results were expressed in terms of odds ratio (OR) and 95% confidence interval (CI). Multivariate logistic regression model was then used as the basis for constructing a nomogram for predicting 28-day mortality in patients with pulmonary collapse.

We then validated the nomogram in the internal cohorts (MIMIC-III split cohorts) and the temporal external cohort (MIMIC-IV). AUC was used to assess discrimination (Yang et al., 2020). SOFA and SAPSII scores within the first-24-h were not recalculated but were directly extracted from the derived database provided by physionet.com. Age+Gender was constructed as a simple logistic model containing only age and gender. AUCs were compared between the nomogram and these comparator models using the DeLong test. We further compared models using ROC-derived sensitivity and specificity, as well as IDI and NRI. Calibration was assessed using calibration curves, Hosmer-Lemeshow tests, calibration slope, and Brier score, and decision curve analysis (DCA) was used to evaluate clinical usefulness. All analyses were performed in R (version 4.0.3; R Foundation for Statistical Computing, Vienna, Austria). A two-sided *P*-value < 0.05 was considered statistically significant.

## Results

### Baseline characteristics

Based on the inclusion and exclusion criteria, 4,088 patients with pulmonary collapse were included from MIMIC-III and MIMIC-IV (1,514 in the training cohort, 650 in the internal validation cohort, and 1,924 in the temporal external cohort). Mortality rates were similar across cohorts (13.0% vs. 13.5% vs. 14.1%, *P* = 0.659). No significant between-cohort differences were observed in gender, age, marital status, race, SOFA score, or ICU length of stay. Baseline characteristics are shown in Table 1.

**Table 1:** Patient characteristics.

Variables	Overall	Training cohort	Validation cohort	External validation cohort	*P*
*N*	4,088	1,514	650	1,924	
Age (year)	66.2 [54.9; 77.6]	64.9 [54.3; 76.3]	66.0 [53.9; 78.0]	66.8 [55.7; 78.2]	0.010
Gender (%)					0.696
Female	1,746 (42.7%)	634 (41.9%)	283 (43.5%)	829 (43.1%)	
Male	2,342 (57.3%)	880 (58.1%)	367 (56.5%)	1,095 (56.9%)	
Marital (%)					0.133
DSW	854 (20.9%)	305 (20.1%)	146 (22.5%)	403 (20.9%)	
Married	1,880 (46.0%)	724 (47.8%)	311 (47.8%)	845 (43.9%)	
Single	1,145 (28.0%)	409 (27.0%)	163 (25.1%)	573 (29.8%)	
Unknown	209 (5.11%)	76 (5.02%)	30 (4.62%)	103 (5.35%)	
Race (%)					0.247
Black	324 (7.93%)	123 (8.12%)	42 (6.46%)	159 (8.26%)	
White	3,130 (76.6%)	1,176 (77.7%)	510 (78.5%)	1,444 (75.1%)	
Yellow	109 (2.67%)	33 (2.18%)	20 (3.08%)	56 (2.91%)	
Other	525 (12.8%)	182 (12.0%)	78 (12.0%)	265 (13.8%)	
Severe score	
GCS	15.0 [14.0; 15.0]	15.0 [14.0; 15.0]	15.0 [14.0; 15.0]	15.0 [14.0; 15.0]	0.001
SAPSII	36.0 [27.0; 45.0]	34.0 [27.0; 44.0]	36.0 [26.2; 45.0]	36.0 [29.0; 45.0]	< 0.001
SOFA	4.00 [2.00; 6.00]	4.00 [2.00; 6.00]	4.00 [2.00; 6.00]	4.00 [2.00; 6.00]	0.137
APSIII	43.0 [33.0; 57.0]	42.0 [32.0; 55.0]	42.0 [33.0; 57.8]	44.0 [33.0; 58.0]	0.008
Laboratory test	
Anion gap(mmol/L)	13.5 [11.5; 15.5]	13.0 [11.5; 15.2]	13.0 [11.3; 15.0]	13.5 [11.5; 15.5]	0.004
Bicarbonate (mmol/L)	24.5 [22.0; 27.0]	24.3 [21.7; 27.0]	24.5 [22.0; 27.0]	24.5 [22.0; 27.0]	0.488
Creatinine (mg/dL)	0.90 [0.70; 1.30]	0.90 [0.70; 1.30]	0.90 [0.70; 1.25]	0.95 [0.70; 1.40]	0.454
Chloride (mmol/L)	104 [100; 108]	105 [101; 108]	105 [101; 108]	104 [100; 108]	0.002
Glucose (mg/dL)	129 [109; 156]	131 [112; 156]	131 [110; 157]	127 [106; 155]	0.009
Hematocrit (g/dL)	31.3 [28.2; 35.3]	31.1 [28.3; 35.0]	31.4 [28.4; 35.1]	31.4 [28.0; 35.5]	0.971
Hemoglobin (g/dL)	10.4 [9.35; 11.8]	10.5 [9.44; 11.8]	10.6 [9.40; 11.9]	10.4 [9.25; 11.9]	0.153
Platelet (K/μL)	216 [157; 295]	214 [155; 293]	225 [163; 304]	215 [156; 293]	0.109
Potassium (mmol/L)	4.10 [3.80; 4.50]	4.12 [3.80; 4.50]	4.15 [3.80; 4.50]	4.10 [3.80; 4.55]	0.653
PTT (s)	31.3 [27.3; 38.8]	31.0 [27.0; 38.2]	31.0 [26.7; 38.3]	31.8 [27.7; 39.6]	0.004
INR	1.25 [1.10; 1.46]	1.27 [1.10; 1.47]	1.23 [1.10; 1.45]	1.25 [1.10; 1.50]	0.330
PT (s)	14.1 [12.9; 15.9]	14.2 [13.1; 15.8]	14.0 [13.1; 15.6]	14.1 [12.6; 16.2]	0.102
Sodium (mmol/L)	138 [136; 141]	138 [136; 141]	138 [136; 140]	138 [136; 141]	0.263
BUN (mmol/L)	19.0 [13.0; 30.0]	18.5 [13.0; 29.0]	19.0 [13.5; 28.0]	19.5 [13.5; 31.0]	0.109
WBC (K/μL)	11.4 [8.59; 15.1]	11.6 [8.64; 15.2]	11.9 [9.10; 15.5]	11.2 [8.30; 14.8]	0.012
Vital signs	
Urine output (mL)	1,549 [986; 2,384]	1590 [1,029; 2,408]	1522 [922; 2,399]	1,520 [964; 2,330]	0.076
Heartrate (bpm)	88.6 [78.0; 100]	88.8 [78.1; 100]	88.4 [77.8; 99.9]	88.6 [78.0; 100]	0.938
Systolic BP (mmHg)	115 [106; 127]	116 [106; 127]	114 [105; 127]	115 [106; 126]	0.155
Diastolic BP (mmHg)	60.3 [54.2; 67.2]	60.0 [53.8; 66.7]	58.6 [53.3; 66.1]	61.0 [54.8; 68.3]	< 0.001
Mean BP (mmHg)	76.0 [69.8; 82.9]	76.4 [70.1; 83.5]	75.6 [69.7; 82.6]	75.8 [69.6; 82.8]	0.140
Respiratory rate (rpm)	19.2 [16.7; 22.0]	18.9 [16.5; 22.0]	19.0 [16.7; 21.8]	19.4 [16.8; 22.2]	0.049
Temperature (°C)	36.9 [36.5; 37.3]	36.9 [36.5; 37.3]	36.9 [36.5; 37.3]	36.9 [36.6; 37.2]	0.574
SpO2 (%)	97.2 [95.6; 98.5]	97.3 [95.9; 98.5]	97.4 [95.9; 98.6]	97.0 [95.3; 98.4]	< 0.001
Comorbidities	
Congestive heart failure (%)					< 0.001
No	3,566 (87.2%)	1,512 (99.9%)	650 (100%)	1,404 (73.0%)	
Yes	522 (12.8%)	2 (0.13%)	0 (0.00%)	520 (27.0%)	
Valvular disease (%)					0.588
No	2,160 (99.8%)	1,512 (99.9%)	648 (99.7%)	0	
Yes	4 (0.18%)	2 (0.13%)	2 (0.31%)	0	
Peripheral vascular (%)					< 0.001
No	3,791 (92.7%)	1,464 (96.7%)	634 (97.5%)	1,693 (88.0%)	
Yes	297 (7.27%)	50 (3.30%)	16 (2.46%)	231 (12.0%)	
Chronic pulmonary (%)					< 0.001
No	3,423 (83.7%)	1,508 (99.6%)	646 (99.4%)	1,269 (66.0%)	
Yes	665 (16.3%)	6 (0.40%)	4 (0.62%)	655 (34.0%)	
Diabetes uncomplicated (%)					< 0.001
No	3,625 (88.7%)	1,514 (100%)	649 (99.8%)	1,462 (76.0%)	
Yes	463 (11.3%)	0 (0.00%)	1 (0.15%)	462 (24.0%)	
Diabetes complicated (%)					< 0.001
No	3,968 (97.1%)	1,509 (99.7%)	648 (99.7%)	1,811 (94.1%)	
Yes	120 (2.94%)	5 (0.33%)	2 (0.31%)	113 (5.87%)	
Liver disease (%)					< 0.001
No	3,768 (92.2%)	1,502 (99.2%)	648 (99.7%)	1,618 (84.1%)	
Yes	320 (7.83%)	12 (0.79%)	2 (0.31%)	306 (15.9%)	
Peptic ulcer (%)					< 0.001
No	4,017 (98.3%)	1,513 (99.9%)	650 (100%)	1,854 (96.4%)	
Yes	71 (1.74%)	1 (0.07%)	0 (0.00%)	70 (3.64%)	
AIDS (%)					< 0.001
No	4,071 (99.6%)	1,514 (100%)	649 (99.8%)	1,908 (99.2%)	
Yes	17 (0.42%)	0 (0.00%)	1 (0.15%)	16 (0.83%)	
Metastatic cancer (%)					< 0.001
No	3,860 (94.4%)	1,486 (98.2%)	646 (99.4%)	1,728 (89.8%)	
Yes	228 (5.58%)	28 (1.85%)	4 (0.62%)	196 (10.2%)	
Solid tumor (%)					0.306
No	3,542 (86.64%)	1,411 (93.2%)	597 (91.8%)	1,534 (79.73%)	
Yes	546 (13.36%)	103 (6.80%)	53 (8.15%)	390 (20.27%)	
Rheumatoid arthritis (%)					0.216
No	2,161 (99.9%)	1,513 (99.9%)	648 (99.7%)	1,854 (96.36%)	
Yes	3 (0.14%)	1 (0.07%)	2 (0.31%)	70 (3.64%)	
Length of ICU stays (day)	11.7 [7.00; 20.2]	11.9 [6.95; 20.3]	11.6 [7.39; 19.3]	11.6 [6.93; 20.2]	0.901
Length of Admission (day)	3.42 [1.96; 7.11]	3.64 [2.02; 7.86]	3.78 [1.98; 7.00]	3.23 [1.91; 6.80]	0.007
Status (%)					0.659
Survival	3,532 (86.4%)	1,317 (87.0%)	562 (86.5%)	1,653 (85.9%)	
Dead	556 (13.6%)	197 (13.0%)	88 (13.5%)	271 (14.1%)	

AIDS, Acquired Immune Deficiency Syndrome; APSIII, Acute Physiology Score III; BUN, Blood Urea Nitrogen; DSW, Divorced/Separated/Widowed; GCS, Glasgow Coma Scale; INR, International Normalized Ratio; PT, Prothrombin Time; PTT, Partial Thromboplastin Time; SAPSII, Simplified Acute Physiology Score II; SOFA, Sequential Organ Failure Assessment; WBC, White Blood Cell.

### Nomogram construction

**Figure 3:**
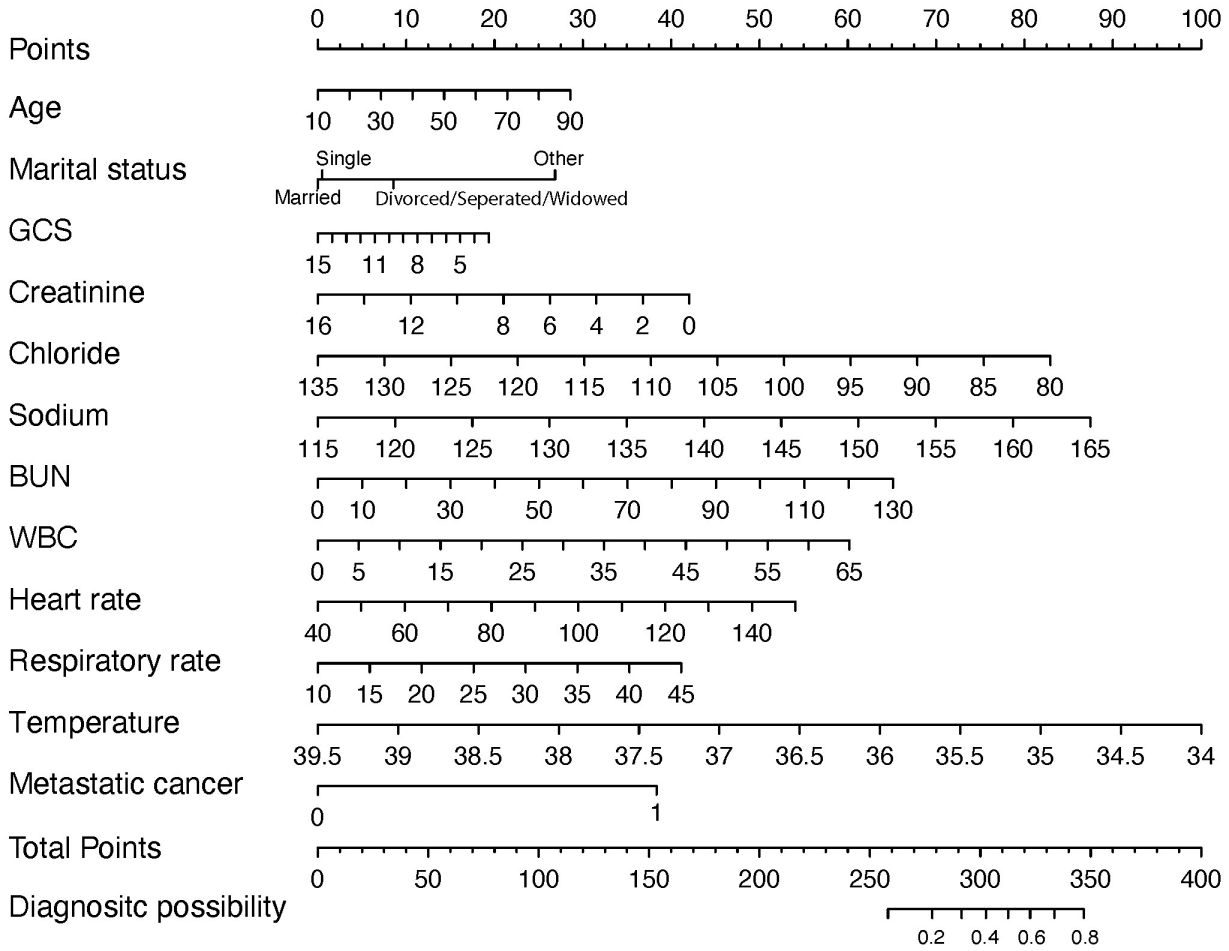
Nomogram for 28-day mortality in pulmonary collapse. The nomogram includes age, marital status, GCS, creatinine, chloride, sodium, BUN, WBC, heart rate, respiratory rate, temperature, and metastatic cancer. Total points are calculated by summing the points for each variable and then mapping the total score to predicted 28-day mortality risk.

LASSO was used for feature selection, followed by multivariable logistic regression. Age, marital status, GCS score, creatinine, chloride, sodium, BUN, WBC, heart rate, respiratory rate, temperature, and metastatic cancer were retained in the final model (Table 2). Higher age, sodium, BUN, WBC, heart rate, respiratory rate, and metastatic cancer were associated with higher 28-day mortality. Married status, higher GCS score, creatinine, chloride, and temperature showed inverse associations. Based on these coefficients, we constructed the nomogram shown in Figure 3. To facilitate clinical interpretation of continuous predictors, odds ratios (ORs) also were re-calculated over conventional increments (e.g., per 10-year increase for age, per 10 bpm for heart rate) using OR^10^ (Table 2).

**Table 2:** Factors independently associated with 28-day mortality in pulmonary collapse patients.

Variables	OR [95% CI]	OR [95% CI] per 10 units	*P*
Age (year)	1.0145 [1.0010–1.0284]	1.1548 [1.0100–1.3170]	0.0370^*^
Marital	
Married	0.6275 [0.4039–0.9800]		0.0390^*^
Severe score	
GCS	0.9372 [0.8868–0.9932]	0.4987 [0.4081–0.9330]	0.0245^*^
Laboratory test	
Creatinine (mg/dL)	0.8205 [0.6758–0.9758]	0.1342 [0.0427–0.7946]	0.0334^*^
Chloride (mmol/L)	0.8547 [0.7374–0.9859]	0.1871 (0.0435–0.8687)	0.0346^*^
Sodium (mmol/L)	1.1888 [1.0267–1.3819]	5.4596 (1.2970–6.1130)	0.0228^*^
BUN (mmol/L)	1.0200 [1.0089–1.0313]	1.2189 (1.0947–1.3576)	0.0000^***^
WBC (K/μL)	1.0384 [1.0132–1.0635]	1.4251 (1.1407–1.8776)	0.0022^**^
Vital signs	
Heartrate (bpm)	1.0158 [1.0027–1.0290]	1.1699 (1.0285–1.3230)	0.0179^*^
Respiratory rate (rpm)	1.0465 [1.0053–1.0891]	1.5811 (1.0547–2.3675)	0.0258^*^
Temperature (°C)	0.5426 [0.4039–0.7237]	0.0035 (0.0000–0.0734)	0.0000^***^
Comorbidities	
Metastatic cancer	6.6554 [2.5930–16.3566]		0.0000^***^

BUN, Blood Urea Nitrogen; GCS, Glasgow Coma Scale; WBC, White Blood Cell; ^*^P < 0.05, ^**^P < 0.01, ^***^P < 0.001. For ease of clinical interpretation, effect sizes for continuous variables are also presented per 10-unit increments using OR^10^.

### Nomogram validation

**Figure 4:**
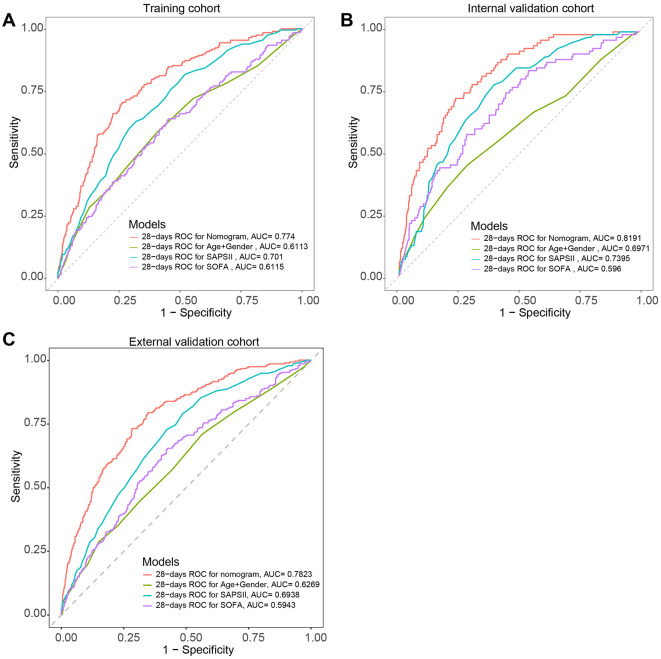
ROC curves for Age+Gender (green), SOFA (purple), SAPSII (blue), and the nomogram (red) in the training **(A)**, validation **(B)**, and external validation **(C)** cohorts.

The C-index of the nomogram was 0.80 (95% CI: 0.77–0.83) in the training cohort, 0.82 (95% CI: 0.78–0.86) in the internal validation cohort, and 0.78 (95% CI: 0.75–0.81) in the external cohort. ROC analysis is shown in Figure 4. The AUCs were 0.774 (95% CI: 0.740–0.808), 0.819 (95% CI: 0.775–0.863), and 0.782 (95% CI: 0.754–0.811), respectively. In all cohorts, the nomogram had higher AUC than Age+Gender, SAPSII, and SOFA. The optimal cutoffs were 0.135 in the training cohort (specificity 0.74, sensitivity 0.71), 0.143 in the internal validation cohort (specificity 0.765, sensitivity 0.727), and 0.132 in the external cohort (specificity 0.716, sensitivity 0.731).

We further compared AUCs using the DeLong test. In the training cohort, the nomogram outperformed SOFA (*P* < 0.001), SAPSII (*P* < 0.001), and Age+Gender (*P* < 0.001). In the internal validation cohort, DeLong tests also favored the nomogram over SOFA (*P* < 0.001), SAPSII (*P* = 0.003), and Age+Gender (*P* < 0.001). In the temporal external cohort, the nomogram had higher AUC than SOFA (*P* < 0.001), SAPSII (*P* < 0.001), and Age+Gender (*P* < 0.001). All DeLong test results indicated statistically significant improvements in discrimination for the nomogram over the comparator models across all three cohorts.

The NRI and IDI were assessed to evaluate the incremental predictive value of the nomogram over three baseline comparators—Age + Gender, SAPSII, and SOFA—across the development, internal validation, and external validation cohorts. As shown in Table 3, all NRI and IDI values were positive and statistically significant (all *P* < 0.01) across all three cohorts and all comparator models. The nomogram demonstrated the improvement in risk reclassification over the Age + Gender baseline, followed by SOFA and SAPSII, a pattern that was consistently observed across the development, internal validation, and external validation cohorts. Similarly, the IDI results confirmed that the nomogram contributed a meaningful and statistically significant improvement in integrated discrimination compared with all baseline models in every cohort. These findings indicate that the nomogram provides generalizable incremental predictive value beyond conventional severity scores and demographic factors alone for the prediction of pulmonary collapse.

**Table 3:** Net Reclassification Improvement (NRI) and Integrated Discrimination Improvement (IDI) of the nomogram compared with baseline models across the development, internal validation, and external validation cohorts.

Metric	Comparing group	Training cohort	Validation cohort	External validation cohort
NRI	Age+Gender	0.615 (0.428–0.754) *P* < 0.001	0.554 (0.365–0.894) *P* = 0.00002	0.594 (0.478–0.764) *P* < 0.001
	SAPSII	0.267 (0.132–0.471) *P* = 0.002	0.357 (0.217–0.702) *P* = 0.002	0.392 (0.264–0.575) *P* < 0.001
	SOFA	0.467 (0.357–0.671) *P* < 0.001	0.470 (0.363–0.818) *P* < 0.001	0.471 (0.354–0.666) *P* < 0.001
IDI	Age + Gender	0.110 (0.086–0.134) *P* < 0.001	0.135 (0.090–0.179) *P* < 0.001	0.124 (0.102–0.147) *P* < 0.001
	SAPSII	0.062 (0.037–0.086) *P* < 0.001	0.095 (0.053–0.136) *P* < 0.001	0.084 (0.063–0.106) *P* < 0.001
	SOFA	0.091 (0.067–0.114) *P* < 0.001	0.116 (0.077–0.155) *P* < 0.001	0.107 (0.085–0.130) *P* < 0.001

**Figure 5:**
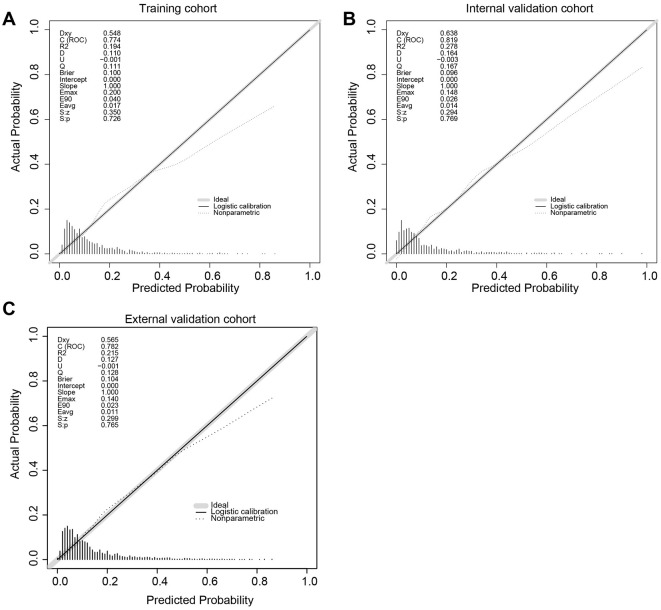
Calibration curves in the training **(A)**, internal validation **(B)**, and external validation **(C)** cohorts.

**Figure 6:**
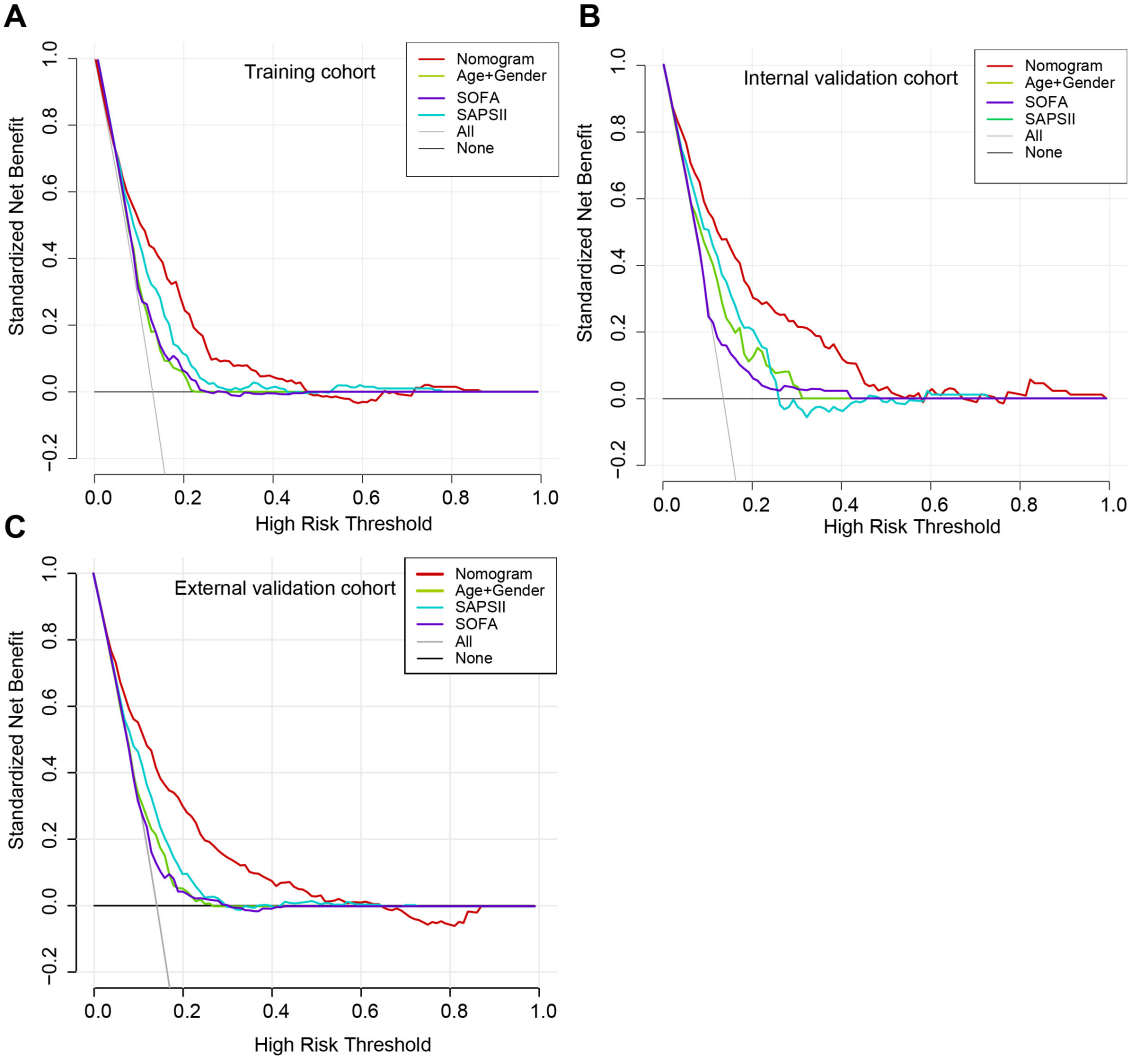
Decision curve analysis comparing clinical net benefit across models in the training **(A)**, internal validation **(B)**, and external validation **(C)** cohorts. Red: nomogram; yellow: Age+Gender; blue: SOFA; green: SAPSII.

Calibration curves (Figure 5) showed visual alignment with the diagonal line, and Hosmer–Lemeshow tests were non-significant in all cohorts (training: *P* = 0.025, validation: *P* = 0.296, external: *P* = 0.407). Brier scores and calibration slopes suggested acceptable overall calibration [training: Brier 0.099, slope 1.000 [95% CI: 0.834–1.165]; validation: Brier 0.095, slope 0.999 [95% CI: 0.778–1.221]; external: Brier 0.103, slope 1.000 [95% CI: 0.862–1.137]]. Additionally, the DCA curves (Figure 6) indicated comparisons with the simple Age+Gender model, SAPSII, and SOFA scoring systems. When the threshold probability was set between 0.1 and 0.4, in all cohorts, the net benefit of clinical intervention following our nomogram was higher than that of the Age+Gender, SAPSII, and SOFA scoring systems.

## Discussion

In this study, we identified factors associated with 28-day mortality in patients with pulmonary collapse using LASSO and multivariable logistic regression, and then built a nomogram. The final model included age, marital status, GCS score, creatinine, chloride, sodium, BUN, WBC, heart rate, respiratory rate, temperature, and metastatic cancer. Across cohorts, the model showed moderate discrimination and higher AUC than Age+Gender, SOFA, and SAPSII. Calibration plots and Hosmer-Lemeshow tests suggested acceptable calibration, but calibration still requires careful interpretation (Van Calster et al., 2019). We treated NRI and IDI as supportive measures, not primary proof of superiority (Kerr et al., 2014). DCA suggested potential clinical value. Overall, the model provides a disease-focused tool for early risk stratification.

The nomogram provides a practical tool for risk stratification at ICU admission. Clinicians can calculate a patient's mortality probability using routinely available variables (age, marital status, GCS, labs, vitals). In clinical use, a higher predicted probability can be used as a trigger for closer monitoring, reassessment of reversible contributors (such as hypothermia, tachycardia, and tachypnea), and earlier multidisciplinary discussion. The model could be integrated into electronic health records for near real-time risk calculation. Because this is a retrospective model with moderate discrimination, it should be used as a decision-support tool rather than a stand-alone basis for treatment decisions.

Pulmonary collapse is associated with serious respiratory complications, and early risk recognition may improve management (Gajic et al., 2011). Early symptoms are often non-specific, and there is no widely used early risk tool tailored to this population (Kewcharoen et al., 2019). Using MIMIC-III and MIMIC-IV, we developed a nomogram to estimate 28-day mortality risk and support early bedside stratification.

As reported in prior studies, aging is associated with structural and functional changes in the lung, including reduced respiratory reserve and gas exchange efficiency (Roman et al., 2016; Xie et al., 2020). This may partly explain why age was associated with higher risk in our model. We also found an inverse association for married status. This may reflect the effect of social support and earlier care-seeking behavior, which has been reported in other disease settings (Pinquart and Duberstein, 2010; Berntsen, 2011). In prognostic modeling, non-biological variables such as marital status can be legitimately included when they improve predictive accuracy, because the goal of a prediction model is risk stratification rather than etiological explanation (Moons et al., 2012; Ramspek et al., 2021). Together, age and marital status capture both biological and social dimensions of risk. GCS reflects neurological status and is commonly associated with mortality risk in critical illness (Gao et al., 2020). In pulmonary collapse, impaired oxygenation may contribute to neurological dysfunction and lower GCS values (Marklin et al., 2021; Lagier et al., 2022). In our model, higher GCS was associated with lower mortality, consistent with this clinical pattern.

Certain predictors exhibited modest per-unit odds ratios (e.g., age, heart rate, BUN) due to their fine-grained measurement scales. To facilitate clinical interpretation, these effects can be re-scaled to reflect clinically meaningful increments without refitting the model (e.g., OR per 10-year increase in age of value 1.10 OR per 10 bpm increase in heart rate of value 1.22). In prognostic prediction modeling, the goal is risk stratification rather than estimation of independent etiological effects; predictors with modest per-unit associations can collectively improve discrimination when combined within a multivariable framework (Steyerberg et al., 2010; Riley et al., 2019). The clinical value of such predictors is therefore assessed by their contribution to overall model performance, rather than by the magnitude of individual coefficients.

We found BUN and creatinine to be independently associated with 28-day mortality. BUN remained a risk marker (OR > 1), while creatinine showed an inverse direction (OR < 1). This apparent “protective” direction for creatinine should not be interpreted as a direct biological benefit of renal dysfunction. In critically ill populations, low creatinine may reflect reduced muscle mass, frailty, or malnutrition, and several studies have reported that low baseline creatinine is independently associated with higher mortality (Cartin-Ceba et al., 2007; Udy et al., 2016). In addition, the association between creatinine and outcomes can be non-linear, and low creatinine values may also be influenced by fluid accumulation and hemodilution, which can mask the severity of kidney injury (Macedo et al., 2010; Hessels et al., 2018). A similar “creatinine paradox” has been reported in severe AKI and AKI-D cohorts, where lower creatinine was associated with worse outcomes (Cerda et al., 2007; Chang et al., 2022). Therefore, in this model creatinine should be interpreted as a contextual prognostic marker in a multivariable framework rather than a causal protective factor.

Pulmonary collapse is often accompanied by lung inflammation (Nguyen et al., 1991; Zeng et al., 2022). WBC is a routine marker of systemic inflammatory response and has been linked to poor outcomes in severe illness (Henry et al., 2020; Chen et al., 2023). In our cohort, higher WBC was associated with higher mortality risk. Heart rate and respiratory rate were also associated with mortality. In pulmonary collapse, hypoxemia can increase sympathetic activity and raise heart rate (Gustman et al., 1983; Paparde et al., 2015). Respiratory rate also tends to rise as a compensatory response to impaired gas exchange (Hudler et al., 2022; Zoccal et al., 2024). In this context, persistent tachycardia and tachypnea may indicate higher physiological stress and worse prognosis. The observed associations with sodium and chloride should be interpreted cautiously. A recent systematic review and meta-analysis of 34 studies (175,021 patients) found that dysregulated serum chloride, particularly hypochloremia, was independently associated with increased mortality in critically ill adults (pooled OR for hypochloremia: 1.55; 95% CI: 1.33–1.81) and may function as a marker of illness severity rather than a direct causal mediator (Wan et al., 2025). We therefore avoid causal inference and interpret these biomarkers as risk-stratification signals in this retrospective dataset.

In addition, body temperature has also been included as a predictive factor for pulmonary collapse. Research indicates that abnormal body temperature is a common presentation among critically ill patients in the ICU, and as body temperature decreases, the mortality rate of patients gradually increases (Garceau et al., 2023). Decreased body temperature may lead to muscle contraction, including bronchial smooth muscle contraction, affecting airway dilation and secretion clearance (Khadadah et al., 2011). This could result in increased airway resistance and retention of secretions in the lungs, further deteriorating the lung function in the affected area and significantly impacting oxygen delivery, rendering the affected lung tissues more fragile (Rubini et al., 2013). In addition, a large observational study using the MIMIC-IV and eICU databases (*n* > 118,000) found that maintaining body temperature at approximately 37 °C was associated with the lowest mortality in critically ill patients, and that increased time spent below 36 °C was associated with higher hospital mortality (Tan et al., 2024). Therefore, maintaining a normal body temperature is crucial in pulmonary collapse for ensuring clear airways and effective oxygen delivery.

Metastatic cancer is an important independent prognostic indicator for predicting mortality, consistent with previous research outcomes. There exists a certain association between metastatic cancer and pulmonary collapse, especially in the late stages of cancer, particularly in lung cancer patients (Godoy et al., 2022). Pulmonary metastases cause pulmonary collapse: metastatic cancer typically refers to primary cancers originating from other sites, such as breast cancer, colon cancer, etc., that have spread to the lungs, forming metastatic lesions (Medeiros and Allan, 2019; Akahane et al., 2023). These lesions may impact lung tissues, leading to the loss of local gas exchange and function in the lungs, resulting in the occurrence of pulmonary collapse. For patients already at an advanced stage of cancer, pulmonary collapse may exacerbate their condition and have an adverse effect on their prognosis. Therefore, the presence of pulmonary collapse may indicate disease progression and a decrease in survival rates.

At present, there is no widely used model designed specifically for 28-day mortality prediction in pulmonary collapse. SOFA and SAPSII are useful general ICU tools, but they were not designed for this disease context (Le Gall et al., 1993; Pandharipande et al., 2009; Lambden et al., 2019). We therefore developed a disease-focused nomogram and compared it with Age+Gender, SOFA, and SAPSII. In our cohorts, the nomogram showed moderate discrimination with statistically significant improvements in AUC over these comparator scores, and higher net benefit on DCA within relevant threshold ranges. This study used public critical care data and standard modeling methods to build a nomogram for 28-day mortality prediction (Balachandran et al., 2015; Wu et al., 2021). By combining routinely available vital signs, laboratory values, and comorbidity data, the model provides a practical estimate of early risk in patients with pulmonary collapse. It is intended to support, not replace, clinical judgment.

However, this study has several limitations. First, disease definition based on the single ICD-9-CM code 518.0 may capture heterogeneous entities (atelectasis, airway obstruction, compression), and misclassification bias may remain. Such administrative mixing can attenuate some predictor effects, worsen calibration in some subgroups, and limit transportability when coding practice or case mix differs across hospitals. We did not perform etiology-based sensitivity or stratified re-analyses because 518.0 alone does not map cleanly to subtypes without added imaging, procedures, or narrative data; inferences therefore apply to patients assigned this code in our cohort, not to every mechanistic subtype of lung collapse. Second, although the model incorporates routinely available ICU variables, it lacks imaging findings (extent of collapse), detailed ventilatory parameters, and directly measured PaO2/FiO2 ratio values (Pandharipande et al., 2009). While SpO2 was available, it is not equivalent to PaO2/FiO2 and cannot fully represent oxygenation severity under varying ventilator settings. Together, these omissions are a major constraint on physiological interpretability: coefficients summarize associations with outcome under routine early ICU data, not a full lung-mechanics or gas-exchange explanation. Third, because this model targets early baseline prediction using first-24-h data, post-baseline treatment variables were not included; this design supports early risk stratification but does not quantify treatment effects on outcome and may leave residual confounding. Fourth, while MIMIC-IV provided temporal external validation, both databases originate from the same institution, representing quasi-external rather than fully independent multi-center validation; validation in other health systems is still needed (Collins et al., 2014). Fifth, we did not perform additional stability analyses (e.g., bootstrap-based variable-selection stability), so residual model instability cannot be excluded, and the exact set of LASSO-retained predictors may not replicate in other samples. Sixth, the moderate class imbalance (13–14% mortality) may affect model performance metrics and threshold-based measures. Seventh, NRI and IDI are supportive and can be sensitive to risk categories; AUC and clinical context should remain primary (Kerr et al., 2014). Finally, prospective validation in diverse clinical settings is needed to assess real-world utility.

## Conclusion

We developed and temporally validated a nomogram for predicting 28-day mortality in ICU patients with pulmonary collapse using routinely available early admission variables. The model showed moderate discrimination with acceptable calibration and may support early risk stratification as a decision-support tool; further external validation in independent health systems is required before broad clinical deployment.

## Data Availability

The original contributions presented in the study are included in the article/supplementary material, further inquiries can be directed to the corresponding authors.
